# Supplementary figures and images for: Deletion of Mediator 1 suppresses TGFβ signaling leading to changes in epidermal lineages and regeneration
Source: PLoS One. 2020 Aug 28;15(8):e0238076. doi: 10.1371/journal.pone.0238076 (PMC7455038; doi:10.1371/journal.pone.0238076)

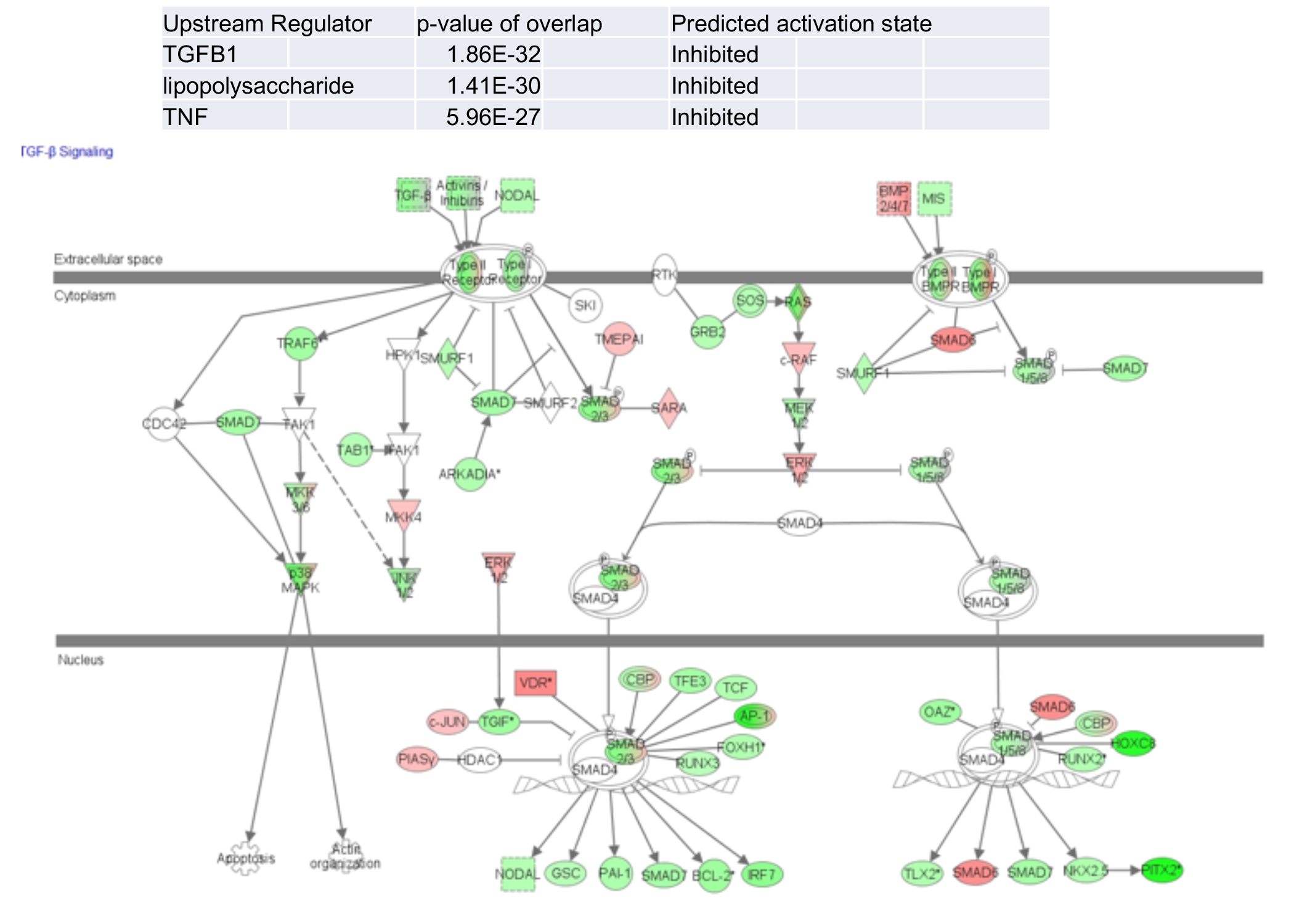

Supplement: S1 Fig — (TIF) [file pone.0238076.s002.tif]

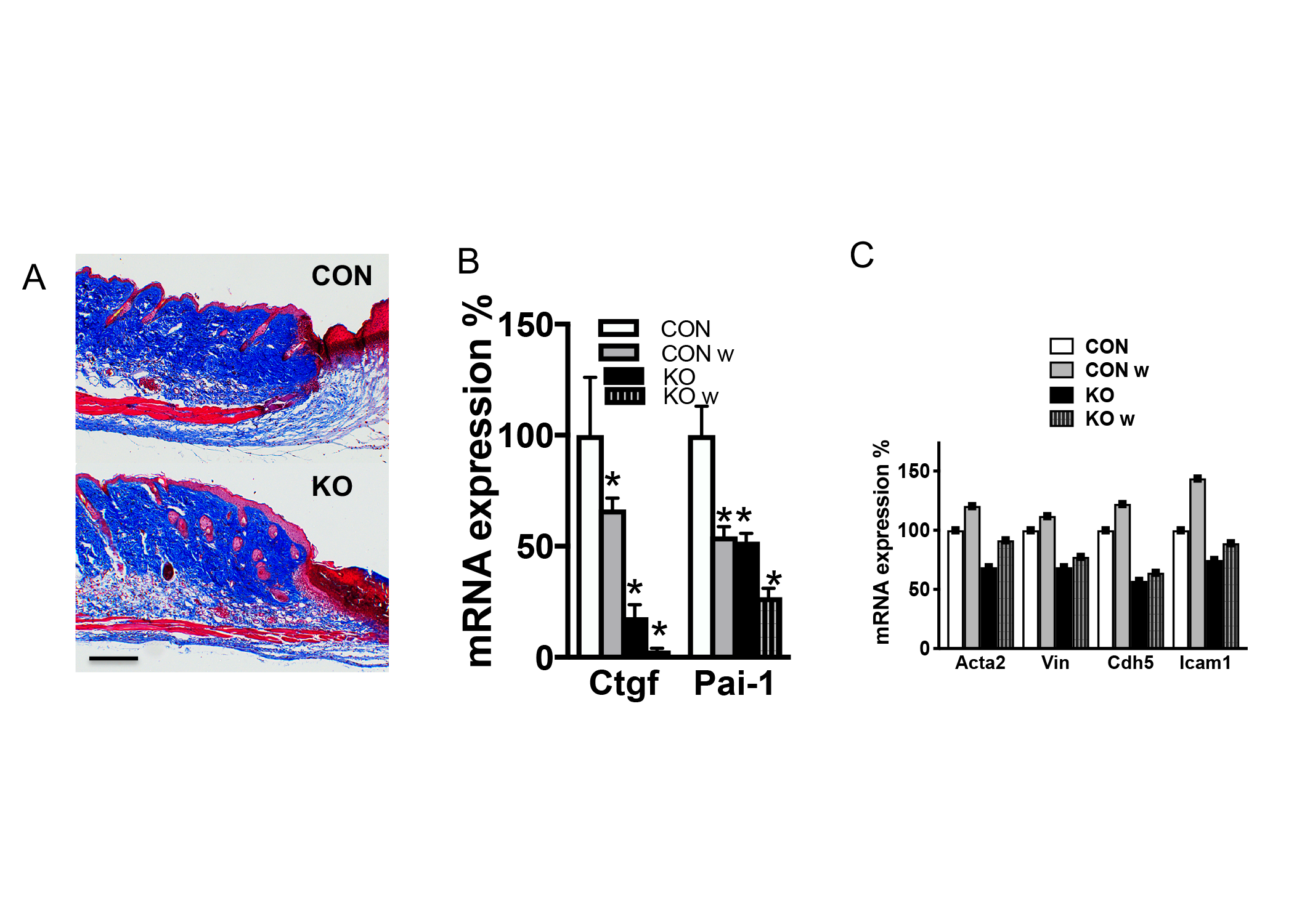

Supplement: S2 Fig — (TIF) [file pone.0238076.s003.tif]

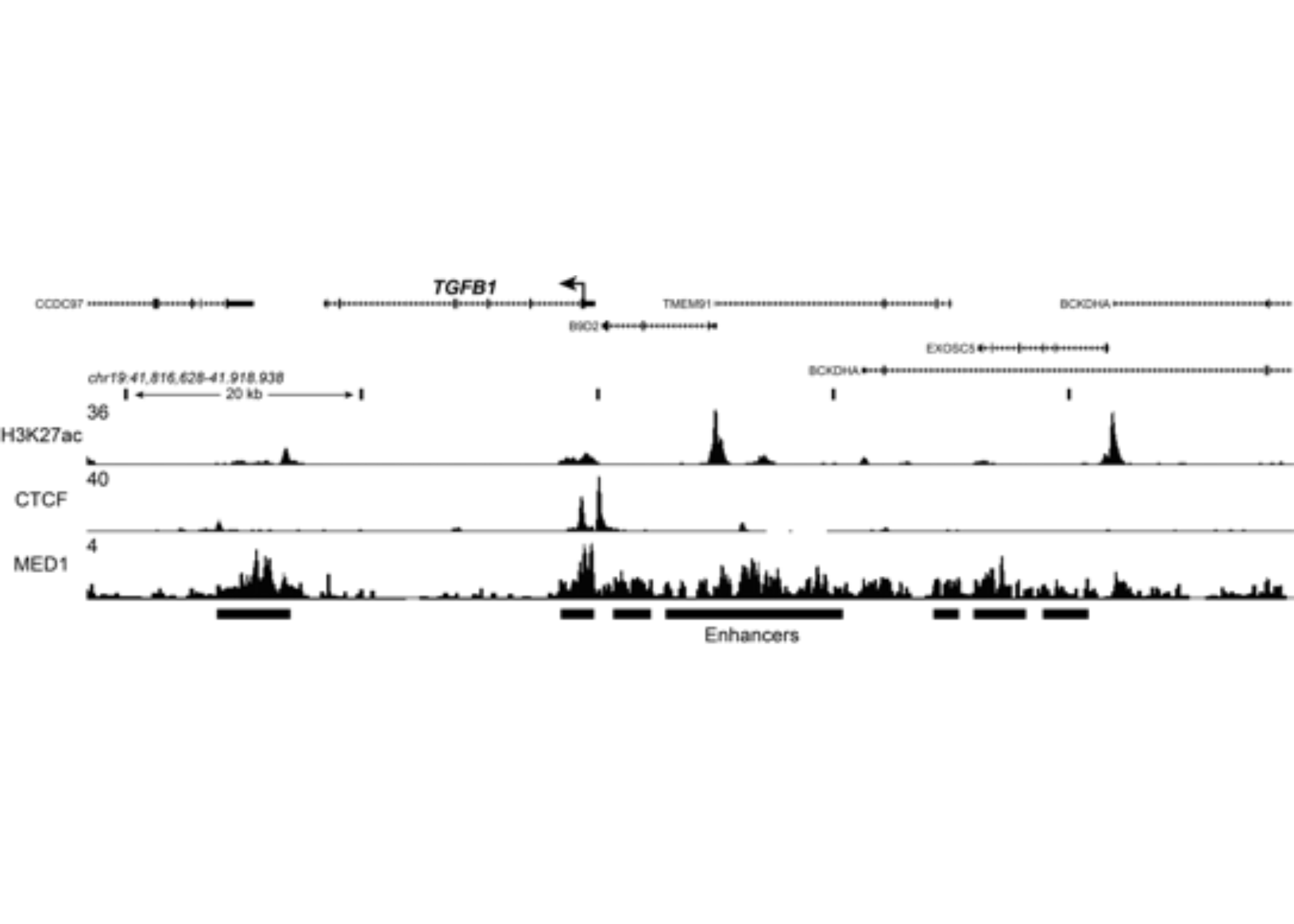

Supplement: S3 Fig — (TIF) [file pone.0238076.s004.tif]

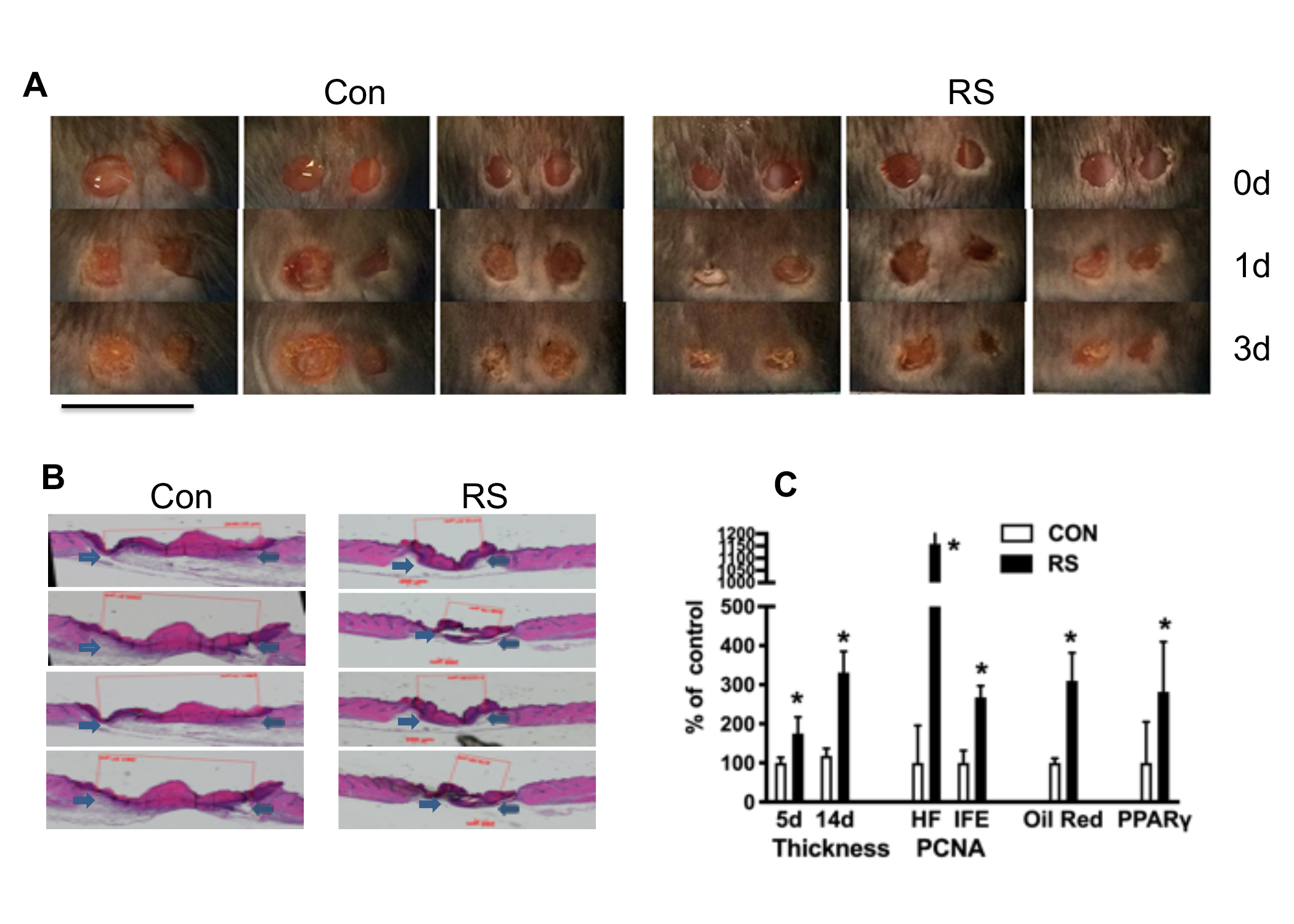

Supplement: S4 Fig — (TIF) [file pone.0238076.s005.tif]
