# Supplementary material for: Deletion of Mediator 1 suppresses TGFβ signaling leading to changes in epidermal lineages and regeneration
Source: PLoS One. 2020 Aug 28;15(8):e0238076. doi: 10.1371/journal.pone.0238076 (PMC7455038; doi:10.1371/journal.pone.0238076)
Supplement: S1 File — (DOCX) [file pone.0238076.s006.docx]

**SUPPLEMENTAL INFORMATION**

**Experimental procedures**

**RNA preparation for microarray analysis**

Total RNA was isolated from epidermal cell preparations, that were isolated from *Med1* KO and CON mice using a Pico Pure RNA purification kit (ABI). RNA was also extracted from skin wounds by Stat60 and purified using RNeasy mini kit (Qiagen). RNAs were analyzed using a Pico Chip on an Agilent 2100 Bioanalyzer (Agilent Technologies) to confirm their purity and concentration. The methods were optimized until a quality of RNA met the standard set by UCLA Genomic Core for Illumina array platform (Mouse Ref-8 v2.0 Ambion).

**Isolation of mouse keratinocytes**

The skin was dissected from *Med1* KO and littermate CON mice. After subcutaneous fat was thoroughly removed, the skin was incubated with 0.25% trypsin/EDTA (Gibco) at 37℃ for 40 min dermis side down. The epidermis was separated from dermis with a scalpel. Keratinocytes were isolated from the epidermis by incubating epidermis with 0.25% trypsin/EDTA at 37℃ for 5 min and gentle pipetting. After neutralizing the trypsin with chelated fetal bovine serum (FBS), undigested tissue was removed through 70 μm and 40 μm cell strainers.

**Real time PCR analyses**

A real time PCR (QPCR) analysis was conducted to validate the microarray results using primers to a subset of genes from the microarray. cDNA synthesis was performed using a TaqMan Reverse Transcription Reagents kit using random hexamer that was provided (Applied Biosystems, N808-0127, Foster City, CA). Real-time quantitative PCR was performed using Power SYBR Green (Applied Biosystems, 1102115) with the 7300 or 7500 Real Time PCR system (Applied Biosystems). Relative mRNA levels compared to the control gene GAPDH were determined using the ΔΔCt method. Primers for the QPCR analysis were derived from the Primer Bank. Primer sets were verified by drawing dissociation curves. Analysis was conducted using at least two independent litters of KO mice or two independent batch of keratinocytes. Averages and standard deviations of relative mRNA expression are calculated. Statistical significance was evaluated by calculating the *p-*values using the Student’s t-test.

**Skin wounding protocol**

Male mice for *Med1* KO and CON (n=3-6 each) were used for wounding studies. They were studied at 8-10 wk of age when CON skin was mostly pink (telogen) as wound healing is related to HF cycling (Ansell et al., 2011). Mice are anesthetized with isoflurane inhalation using vaporizer. After shaving hair, two 3 mm full thickness skin biopsies were taken from the upper portion of their backs. Respiratory rate is monitored and adequacy of anesthesia will be confirmed by lack of response to foot pinch or whisker stimulation during the procedure. After surgery, mice are kept in warm pads for their recovery from anesthesia. All efforts were made to minimize suffering. As KO skin always contained abnormal black/grey anagen patches mixed in pink telogen skin, skin biopsies were taken from both anagen and telogen skin only in the case of KO mice. The hair cycle was judged by visual inspection of skin color. The wounds were monitored during their recovery as described (Oda et al., 2015). Skin wounds (3mm) were harvested after 1 or 3 d of wounding following euthanasia done by decapitation under overdose of isoflurane. All the mice are sacrificed after wounding studies and are not use for other purposes. For analyses, one of the wounds was used for histology and the other for mRNA levels. The rim of tissue around the other wound in the same mouse was harvested and stored in RNA later for subsequent mRNA expression analyses.

**Histological analysis**

*Med1* KO and littermate control (CON) skin or RS treated skin were fixed by 4% PFA. They were dehydrated and embedded in paraffin. The skin was sectioned at 7 mm at angles to show whole hair follicles. The centers of wounds were also sectioned in the same way. They were stained with hematoxylin and eosin (HE) for morphological analyses and for measurement of distances between wound edges to determine rates of re-epithelialization.

**Morphometric measurement of re-epithelialization.** Photographs with a ruler were obtained at each time point, digitized, and areas and perimeters of the epithelial margins measured with software in Bioquant. Measurements across the wound as well as between the leading edges of the epidermis were measured and expressed as a ratio of the distance between the leading edges of the epithelial tongues to the overall diameter of the wound as re-epithelialization rate.

**Immunostaining**

Paraffin embedded skin sections were treated with antigen unmasking solutions (Vector lab Citrate-based, H-3300). They were blocked by an avidin/biotin blocking kit (Vector lab, SP-2001), and incubated with primary antibodies against Krt1 (Covance), involucrin (Covance), filaggrin (Covance), loricrin (Covance). They were then developed by Vectastain Elite ABC kit (Vector Lab) using DAB staining solution (Vector Lab, SK-4100) until brown signals were visible. The sections were counterstained with Gill’s Hematoxylin.

**Immunofluorescence**

Non-fixed skin samples were dehydrated by 30% sucrose and embedded in OCT compounds. Frozen sections were prepared and blocked with 5% fetal bovine serum (FBS), 1% bovine serum albumin (BSA), 0.01% Tween 20, and incubated with Krt15 (Covance, chicken antibody), PPARγ (Santa Cruz Biotech H-100 sc-7196), TGFβ1 (V) (Santa Cruz sc-146), and CTGF (Santa Cruz Biotech, L-20), and COL6A1 (Santa Cruz). Cultured keratinocytes were fixed by 4% PFA for 10 min and incubated with antibodies against Santa Cruz sc-146 and p-SMAD2/3 (Phospho-SMADd2 (Ser465/467)/SMAD3(Ser423/425) (Cell Signaling D27F4 mAb #8828). They were subsequently incubated with species specific secondary antibodies conjugated with fluorescent dyes (Invitrogen Molecular Probes), including Alexa 594 (red) and Alexa 488 (green). Cells were counterstained by b-catenin (Santa Cruz E-5). They were then counterstained with DAPI. The images were taken and merged through multi-dimension analysis (add software name Carl Zeiss).

**PCNA staining on skin section**

The skin sections were stained with a PCNA staining kit (Invitrogen) according to the manufacturer’s instructions with minimum exposure of substrate in order to specifically and quantitatively detect highly proliferative cells. The PCNA positive cells were quantitated by image analysis using Bioquant. The percentage of PCNA positive cells in KO compared to CON was calculated. Statistical significance was evaluated by counting cells in 6 or more sections in each group each with 3 mice/group.

**Primary keratinocyte culture**

Epidermis was separated from neonatal human foreskin and primary keratinocytes were isolated from epidermis and cultured in serum-free keratinocyte growth medium 154CF containing growth supplements (Gibco, Cascade biologics) containing low (0.07 mM) calcium. Second to fourth passages of keratinocytes were cultured with same medium in low calcium to maintain them. Cells were used under pre-confluent conditions in which density dependent epidermal differentiation was avoided.

### siRNA silencing of *MED1* in primary keratinocytes

Second or third passage of primary keratinocytes were plated in 12 well plates at a density of 50-70% confluency on the day of transfection. Cells were transfected with either siRNA for *MED1* (Thermo, Dharmacon, ON-TARGET Plus L-040964-01) or control siRNA (Thermo Dharmacon D-0012206-13-05). A pool of four siRNA duplexes with UU overhangs and a 5′ phosphate on the antisense strand specific for human *MED1* (SMART pool PLUS M-004267-00 5′-GGTCTGATTTGGTTAAGAA-3′, 5′-GTTCAGAGATCATAGGAAT-3′, 5′-TAAATAGGGTTCAGAGATC-3′, 5′-CAAGCTGGGTGAATTAGAA-3′) Dharmacon Research (Lafayette, CO) were used. Cells were transfected with siRNA using siLentFect (Bio-Rad) according to the manufacturer’s protocol. The blocking efficiency of *MED1* was confirmed by qPCR analysis.

**EdU Cell proliferation assay**

Cultured keratinocytes maintained in 154CF with 0.07mM were labeled with 10 uM EdU for 60 min. Incorporated EdU was visualized by Click-iT EdU Alexa Fluor^TM^ 594 imaging kit (Thermo Scientific) and observed with DAPI counter staining. The numbers of EdU positive cells per total cells were determined in at least 3 fields using two independent batch of primary cultures of keratinocytes.

**Cell migration *in vitro* assay**

Confluent keratinocyte cultures were switched to 154CF media without supplements to stop their proliferation for 4 hr. The cultures were scratched using a 20 ul pipette tip (Rainin).  Cell migration was monitored by taking photographs in the same field before and after (16 hr) scratching at 5 or more different locations.  Cell migration was assessed by measuring the unoccupied space using Bioquant software. Statistical significance was evaluated by t-test using at least 5 different fields in two independent batches of keratinocytes.

**Recombinant TGF**β**1 treatment**

Recombinant mouse TGFβ1 (R&D system) was activated by reconstituting in 4 mM HCl containing at least 0.1% bovine serum albumin. Transfected cells were treated with different concentrations of rTGFβ1 in the supplement free media overnight. The study of human cells was conducted with the approval of the VA safety committee.

**Inhibition of TGF**β **signaling by RepSox**

Small molecule TGFβ inhibitor, RepSox (2-[3-(6-Methyl-2-pyridinyl)-1H-pyrazol-4-yl]-1,5-naphthyridine, Sigma) was dissolved in 50% propylenge glycol (PPG), 10% EtOH or in acetone. A volume of 30 ul (0.6 μmole) of RepSox or vehicle were applied daily for 5 days on a marked site of back skin of C57BL6 mice (male) at 8-10 wk of age. Full thickness biopsies (3 mm) were taken from drug pretreated or vehicle treated skin, and wound healing was monitored by the same methods as previously performed [28].

**Figure legends for Supplemental figures.**

**S1 Table**  **TGFβ1 is listed as an upstream regulator for *KO keratinocytes***. Upstream regulators are listed by IPA software, which are potentially responsible for the observed changes in Med1 KO keratinocytes compared to CON. The name of regulator, fold change of the regulator, molecule type, predicted state (activated or inhibited), z-score, statistical significance (p-value) are calculated by IPA software. TGFβ1 is the most significant regulator shown by red color, and wnt/β-catenin is also shown (blue).

**S1 Fig**  **TGF**β **signaling pathway is inhibited in KO wounded skin (1d).**

Upper panel: TGFβ1 also is listed as an inhibitory upstream regulator responsible for changes in KO wounded skin at 1 day after wound injury.

Lower panel: IPA software applied microarray data (KO and CON skin wounds at 1 d) to their template for TGFβ signaling, in which many genes were down-regulated (green). Subcellular localization (extracellular, cytoplasm, and nucleus) of their ligands, receptors, and regulatory genes, *Smad* mediated transcription, and TGFβ downstream target genes are shown. Their relationships are shown by arrows (activation) and stop signs (inhibition).

**S2 Fig Med1 deletion affects wounded skin 1 day after injury.**

(A) Masson trichrome staining at wounding edge. Representative images of three wounds (n=3) from CON and KO, in which collagen depositions in dermis is shown by blue color (Bars = 200 μm).

(B) The mRNA levels for epidermal TGFβ targets in non-wounded (CON, KO) and wounded skin (CON w, KO w) at 1d after injury. The percentage compared to non-wounded CON skin is shown (qPCR, mean +/- SD n=3, t-test, *p<0.05).

(C) The mRNA levels of TGFβ regulated myofibroblast markers, α-smooth muscle actin (α-SMA acta2), vinmentin (vin), VE-cadherin (Cdh5), intercellular adhesion molecule 1 (Icam1) as shown in (B) (1d) (microarray, average of 3 wounds).

**S3 Fig MED1 forms large enhancer at the TGFβ1 loci**.

ChIP-seq binding profiles for H3K27ac, CTCF, and MED1 in keratinocytes. The black bar shows a large enhancer, in which MED1 is densely incorporated. The profile was reproduced in two different batches of primary keratinocytes, and typical profile is shown.

**S4 Fig 5 TGF**β**1 inhibitor RepSox accelerates wound re-epithelialization rate**.

(A) Photographs of skin wounds at 0d, 1d and 3 d after 3 mm biopsies were taken from back skin that were treated with vehicle (CON) and RepSox (RS) (2 wounds in 3 mice each group). (B) HE stain of skin wounds after 3 days. Blue arrows show epithelial edges to close the wounds. Red line is the distance of two edges to calculate the re-epithelialization rate. (C) Quantification of histologic images shown in Fig. 5, in which epidermal thickness (Fig. 5A), PCNA staining (Fig. 5C) and PPARγ positive cells (Fig. 5F) were quantitated by Bioquant software. Percentiles of RS treated skin compared to vehicle control skin (CON) were calculated. Significance was calculated by t-test (n=6 *p<0.05).
